# Protein Requirements for Maximal Muscle Mass and Athletic Performance Are Achieved with Completely Plant-Based Diets Scaled to Meet Energy Needs: A Modeling Study in Professional American Football Players

**DOI:** 10.3390/nu16121903

**Published:** 2024-06-17

**Authors:** David M. Goldman, Cassandra B. Warbeck, Micaela C. Karlsen

**Affiliations:** 1 Department of Public Health, University of Helsinki, 00014 Helsinki, Finland; 2 Department of Research and Development, Metabite Inc., New York, NY 10036, USA; 3 Department of Family Medicine, University of Alberta, Edmonton, AB T6G 2R3, Canada; cwarbeck@ualberta.ca; 4 Department of Research, American College of Lifestyle Medicine, Chesterfield, MO 63006, USA; mkarlsen@lifestylemedicine.org; 5 Departments of Applied Nutrition and Global Public Health, Adjunct Faculty, University of New England, Biddeford, ME 04005, USA

**Keywords:** American football, National Football League, athletic performance, muscle mass, plant-based, sports nutrition, protein, leucine

## Abstract

American football players consume large quantities of animal-sourced protein in adherence with traditional recommendations to maximize muscle development and athletic performance. This contrasts with dietary guidelines, which recommend reducing meat intake and increasing consumption of plant-based foods to promote health and reduce the risk of chronic disease. The capacity of completely plant-based diets to meet the nutritional needs of American football players has not been studied. This modeling study scaled dietary data from a large cohort following completely plant-based diets to meet the energy requirements of professional American football players to determine whether protein, leucine, and micronutrient needs for physical performance and health were met. The Cunningham equation was used to estimate calorie requirements. Nutrient intakes from the Adventist Health Study 2 were then scaled to this calorie level. Protein values ranged from 1.6–2.2 g/kg/day and leucine values ranged from 3.8–4.1 g/meal at each of four daily meals, therefore meeting and exceeding levels theorized to maximize muscle mass, muscle strength, and muscle protein synthesis, respectively. Plant-based diets scaled to meet the energy needs of professional American football players satisfied protein, leucine, and micronutrient requirements for muscle development and athletic performance. These findings suggest that completely plant-based diets could bridge the gap between dietary recommendations for chronic disease prevention and athletic performance in American football players.

## 1. Introduction

Dietary protein is a primary concern for many American football players [1]. The majority of football players believe that consuming additional protein is likely to improve their strength and speed [2] and is necessary for muscle growth and development [3]. Many football players and other athletes also believe that protein is the main source of energy for exercising muscles [4] and preferentially select high-protein foods such as meat because they believe their bodies require them to remain healthy [1]. In contrast, many football players consider carbohydrates to be of secondary importance [1], which is reflected in their under-consumption relative to sports nutrition recommendations [5,6].

The International Society of Sports Nutrition supports the consumption of animal-based protein sources on account of their high content of indispensable amino acids (IAAs) for muscle development [7], which is an important gauge of the physical performance required to succeed in the National Football League (NFL) [8,9]. Sample meals developed for NFL players emphasize animal protein as well [10]. This guidance is apparent in the diets of competitive football players, which typically include large quantities of meat and other sources of animal protein [6,11,12,13]. Cardiovascular diseases (CVDs) are the leading cause of death among former NFL players [14], and higher intake of animal protein has been shown to increase the risk of CVD mortality, whereas higher intake of plant protein is inversely associated with CVD mortality [15,16]. Substituting protein-rich foods from animal sources with plant sources has therefore been shown to reduce risk of CVD and premature death [17,18,19]. National dietary guidelines recommend that young men decrease their intake of meat, poultry, and eggs and increase their intake of legumes and whole grains to reduce the risk of chronic diseases including CVD. Similarly, clinical practice guidelines commonly recommend reducing meat consumption and increasing consumption of legumes and whole grains to reduce CVD risk [20]. The suggested dietary guidelines for reducing the risk of CVD and premature death seem at odds with the goal of enhancing muscle development and athletic performance in American football players. While disease risk among American football players has been understudied, this disparity implies these athletes may face a dilemma, having to decide between long-term health and achieving success in their sport.

Concurrently, interest in plant-based diets for health and athletic performance has been growing throughout the past decade [21]. Plant-based diets emphasize fruits, vegetables, whole grains, legumes, nuts, seeds, and plant products and limit or eliminate animal-based foods [22]. In this study, the term “completely plant-based diet” refers to any diet that consists of predominantly whole, plant-foods, is devoid of animal products, and may include processed plant foods to the extent that fiber intake still meets or exceeds recommendations [23]. Traditional beliefs that plant-based diets are suboptimal for athletic performance and muscular development are pervasive, with commonly cited issues including inadequate provision of total protein and/or specific amino acids such as leucine [24,25,26]. Leucine is uniquely valuable for muscle and strength development because of its dual functionality as a building block for protein and as a potent stimulator of muscle protein synthesis (MPS), which is the major facilitator of nutrient-induced muscular hypertrophy [27]. Sources of plant-based protein have also been characterized as containing low quantities of lysine and methionine [28], although limited research suggests that adults who follow completely plant-based diets while undertaking resistance exercise training (RET) do not demonstrate inadequate intakes [29].

The research team previously modeled dietary values in bodybuilders pursuing maximal muscular hypertrophy while consuming plant-based diets [23]; this study extends this research into the sport of American football. We aimed to evaluate the accuracy of the belief that athletes who adhere to plant-based diets should consume protein supplements [30,31,32]. The objective of this analysis is therefore to assess whether completely plant-based diets, scaled to meet the caloric needs of American football players, provide sufficient protein and leucine to maximally support muscular hypertrophy, strength, and athletic performance while meeting micronutrient needs.

## 2. Materials and Methods

This analysis closely follows methods that have been previously described and used to model protein and leucine values in male bodybuilders [23]. Briefly, dietary data were derived from the Adventist Health Study 2 (AHS-2) because that study included a large population following a completely plant-based (vegan) diet (*n* = 5694) with mean fiber intakes that surpassed recommendations (46.7 versus 19–38 g/day [33,34]. The study used an FFQ that surveyed intake of foods, beverages, and dietary supplements. Intakes were reported as the sum of dietary and supplement provisions, and respective intakes from these sources were not differentiated [33]. Energy requirements were derived from a report on NFL athletes [10] in which values were calculated using the Cunningham equation, which has been validated in athletic populations [35,36].

The protein intake level required by American football players to maximize athletic performance was determined through consulting the sports nutrition textbook published by the Sports, Cardiovascular, and Wellness Nutrition Dietetics Practice Group of the Academy of Nutrition and Dietetics. This handbook for professionals indicates that 1.4–1.7 g of protein per kilogram of body mass per day (g/kg/day) is recommended for football players [37]. This range aligns with the International Olympic Committee (IOC) consensus statement that the optimal and recommended protein intake for high-performance athletes pursuing maximal strength and muscular hypertrophy is 1.6 g/kg/day [38]. Protein requirements were therefore determined as 1.6 g/kg/day, which reflects previously published recommendations for American football players [10,11,39]. Total protein intake in the AHS-2 cohort following a vegan diet was reported as 14.5% of total calories [33]. Applying this percentage to mean energy requirements for each player position provided absolute protein values. Dividing these values by mean body mass yielded relative protein values in g/kg/day.

The methods used to determine leucine requirements have been previously described [23]. Consistent with this, 2 g/meal of leucine at four meals per day, totaling ≥8 g/day, was established as the target. The leucine contents of various commonly consumed animal- and plant-sourced proteins were originally derived from the Food and Agriculture Organization (FAO), the United States Department of Agriculture (USDA), and other sources [40,41,42,43,44,45,46,47,48,49]. These items included beef, egg, and cod for animal proteins, milk, whey, and casein for dairy proteins, and lentils, quinoa, black beans, corn, soy, peas, rice, oats, hemp, potatoes, wheat, spirulina, and mycoprotein for plant proteins [50,51]. The mean leucine contents as a percentage of total protein (±SEM) for animal, dairy, and plant proteins were 8.8% ± 0.7%, >10%, and 7.1% ± 0.8%, respectively [52]. The mean value for plant foods multiplied by total dietary protein was used to determine leucine contents.

The modeling of micronutrients and other key nutrients has been described previously [23]. Target levels for saturated fat, total omega-3, linoleic acid, fiber, vitamin A, vitamin B6, folate, vitamin B12, vitamin C, vitamin D, vitamin E, calcium, iron, magnesium, phosphorus, potassium, sodium, and zinc were set based on the dietary reference intakes (DRIs) [53]. Tolerable upper limits (ULs) were set based on established recommendations from the National Academy of Medicine, formerly the Institute of Medicine [54,55,56,57]. Essential nutrients for which values were not available were thiamin, riboflavin, niacin, pantothenic acid, biotin, chloride, iodine, sulfur, cobalt, copper, manganese, and selenium. Values for adult men aged 19–30 were selected for comparison with projected values in scaled plant-based diets, because these demographics encompass the majority of players profiled in the NFL [8].

## 3. Results

### 3.1. Energy Requirements

Daily energy requirements for different positions varied as follows: defensive lineman (DL) (6250 kcal), offensive lineman (OL) (6350 kcal), running back (RB) (5850 kcal), tight end (TE) (6150 kcal), linebacker (LB) (6050 kcal), and quarterback (QB) (5300 kcal), as shown in Table 1.

### 3.2. Protein Requirements and Levels

**Figure 1:**
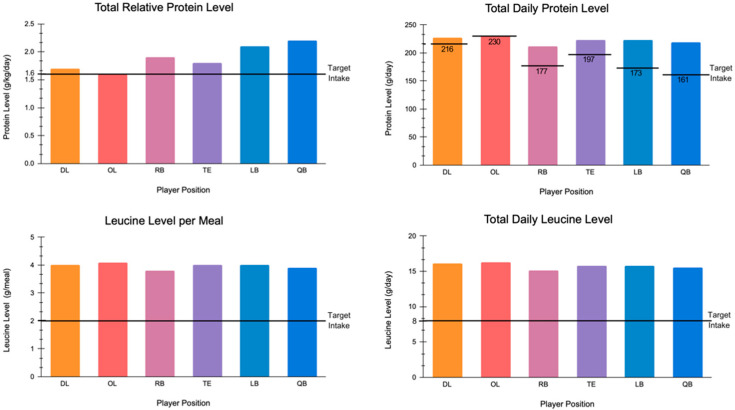
Protein and leucine levels scaled to meet the energy requirements of American football players competing in the National Football League following completely plant-based diets, in relation to thresholds proposed to maximize gains in muscle mass and strength in response to resistance exercise training. DL: defensive lineman; OL: offensive lineman; RB: running back; TE: tight end; LB: linebacker; QB: quarterback.

Protein values in g/kg/day varied as follows: DL (1.7), OL (1.6), RB (1.9), TE (1.8), LB (2.1), and QB (2.2), as presented in Table 1. Protein values exceeded requirements for maximal muscular hypertrophy and athletic performance, as shown in Figure 1.

### 3.3. Leucine Levels

Modeled leucine levels in g/day varied as follows: DL (16.1), OL (16.3), RB (15.1), TE (15.8), LB (15.8), and QB (15.5). Modeled leucine levels in g/meal varied as follows: DL (4.0), OL (4.1), RB (3.8), TE (4.0), LB (4.0), and QB (3.9). These values are shown in Table 1. Modeled levels relative to recommendations are shown in Figure 1.

### 3.4. Other Macronutrients

Mean saturated fat levels (5% of calories) were within recommended limits (10% of calories). Fiber levels (124–146 g/day) exceeded targets (74–89 g/day). Results are shown in Table 2.

### 3.5. Key Micronutrients

Projected diets for each player position surpassed all recommended micronutrient targets for adequate intake. There were seven micronutrients that exceeded the UL for all player positions: folate (2353–2819 vs. 1000 mcg/day), calcium (3063–3670 vs. 2500 mg/day), iron (84–100 vs. 45 mg/day), magnesium (1728–2070 vs. 350 mg/day), zinc (43–52 vs. 40 mg/day), and sodium (9357–11,211 vs. 2300 mg/day). Phosphorus intake marginally surpassed the UL for every position (4010–4353 vs. 4000 mg/day) except quarterback (3633 mg/day). Results are shown in Table 2.

## 4. Discussion

This study examined the adequacy of completely plant-based diets, scaled to the energy requirements of NFL players, in achieving the recommended levels of protein, leucine, and micronutrients to support athletic performance, muscular hypertrophy and strength, and nutritional needs. Although supplementary protein provisions in these modeled diets were limited [59], data pertaining to the extent to which other supplements (e.g., multivitamins) were included were unavailable. However, it is recommended that individuals who follow plant-based diets consume vitamin B12 from supplements or fortified foods [60]. Our findings indicate that these diets met or exceeded protein recommendations for maximizing athletic performance and gains in muscle mass and strength (1.6 g/kg/day) through providing 1.6–2.2 g/kg/day protein [10,11,38,39]. Results also indicate that the scaled, completely plant-based diets exceeded leucine requirements for the maximal stimulation of MPS (2 g/meal) [61,62,63] through providing 14.2–16.4 g/day, or 3.8–4.1 g/meal at each of four daily meals. 

The results of this research have important implications for both public health and athletic sectors. They indicate the potential for alignment between guidelines for preventing and treating chronic diseases and the promotion of muscle mass, strength, and athletic performance in American football players, and linemen in particular, for whom CVD poses a significant risk. Modeling indicates that American football players can fulfill their dietary needs for optimal athletic performance simply through consuming larger portions of completely plant-based meals.

Calorie requirements used for this study (5300–6350 kcal/day) were similar to or greater than intakes reported in other samples of American football players. Cole et al. (2005) had 30 National Collegiate Athletic Association (NCAA) Division 1 football players complete three-day food records, and reported mean energy intakes were 3288 kcal/day [6]. Abbey et al. (2017) instructed 88 NCAA Division 3 football players to complete FFQs, and estimated mean energy intakes were 5225 kcal/day [11]. The former study included players of all positions and the latter exclusively reported intakes in linemen.

Energy requirements are largely determined according to body mass and composition as well as activity level [36,64], which vary significantly between athletes of different positions [8,13,65,66,67,68,69]. Calorie requirements and intake levels therefore exhibit significant differences between American football players [6,11,65]. For example, defensive ends typically have lower levels of body mass than offensive linemen, and quarterbacks have lower lean body mass and physical activity levels than running backs, contributing to their lower calorie requirements [10]. Furthermore, player position determines the predominant energy system used by American football players, with linemen relying more heavily on anaerobic metabolism compared with linebackers and defensive backs, for whom aerobic metabolism is more consequential [10]. Phosphocreatine supports energy generation for anaerobic metabolism and fatty acids provide energy for aerobic metabolism, while glycogen is central to both energy systems and is therefore a fundamental substrate for high-intensity physical performance [10,70,71]. Plant-based diets are high in carbohydrates, which have been proposed to confer performance-enhancing effects in athletes [72].

The significant heterogeneity in anthropometrics and activity levels in American football athletes may also be responsible for the observed variance in reported energy values. Self-reported dietary assessments in athletes have also been shown to underestimate energy intake by 19% [73], suggesting that actual intakes may have more closely approximated those used in our study. Kirwan et al. (2012) assessed the dietary habits of 15 NCAA Division 1 football players using three-day food records and reported mean early season and postseason intakes of 3518 and 5115 kcal/day, respectively [39], indicating that the temporality of data collection can also influence dietary variability. A previous study evaluated the diets of 44 Polish American football players using three-day dietary recalls and determined that offensive and defensive players consumed on average 2472 and 3086 kcal/day, respectively. The authors reported that these levels were insufficient to meet athletes’ needs [12].

Similarly, an assessment of the diets of 185 Turkish collegiate American football players used three-day diet records and calculated their mean energy intake to be 2750 kcal/day [5]. The low energy intakes reported in that study may have resulted, in part, from lower levels of lean body mass (LBM), which was 27–30 kg lower in Turkish versus NFL linemen [5,65] and is central to the Cunningham equation used to calculate energy needs [74]. Therefore, the calorie range used for our calculations exceeded reported intakes in Polish and Turkish American football players, as well as NCAA athletes in the early season, but was similar to reported values in NCAA linemen and other positions during the postseason.

Protein values modeled in this study were 1.6–2.2 g/kg/day, which meet or exceed recommended levels (1.6 g/kg/day) for American football players [10,37,38]. These values also align with recommendations for bodybuilders [75], and larger intakes do not promote additional RET-induced gains in muscle mass and strength [76]. Dietary survey data in American football players are limited, but previous work has found that NCAA Division 1 football players exceeded dietary protein recommendations, consuming 1.8–2.3 g/kg/day [39]. Animal-sourced foods probably comprised the majority of protein consumed, as reflected in the mean dietary cholesterol and fiber intakes that ranged from 757 to 1004 mg/day and 18 to 19 g/day [39], exceeding and falling below recommended levels [34] and representing animal and plant protein intake, respectively [77,78,79]. Similarly, others assessed dietary intakes in NCAA Division 3 linemen, whose mean protein intake of 2.0 g/kg/day exceeded recommendations. The majority of athletes reported consuming meat and dairy products on a daily basis (52.3% and 82.8%, respectively), while less than half of the athletes ate fruits or vegetables each day (47.1% and 38.4%, respectively) [11].

Protein requirements may be met less consistently in populations beyond the United States. The previous analysis found that mean protein intakes in Polish American football players met requirements in offensive unit athletes but not in defensive unit athletes, reaching 1.6 and 1.4 g/kg/day, respectively. These athletes consumed approximately triple the amount of animal protein relative to plant protein, reflected in intakes of animal and plant protein of 94–113 g/day and 30–39 g/day, respectively [12]. Survey responses from adult Polish American football players also reported daily consumption of meat and cold cuts in 67% of athletes, egg consumption several times per week in 90% of athletes, and legume consumption several times per month in only 45% of the sample [13]. Moreover, protein intakes in Turkish American football players generally did not reach recommended levels. Mean protein intake was 1.55 g/kg/day, and only 39.5% of the sampled athletes achieved protein intakes of ≥1.6–1.7 g/kg/day. Mean cholesterol and fiber intakes were in the ranges 564–598 mg/day and 20–24 g/day, respectively, suggesting that the diets of these athletes were also predominantly animal-based [5].

In these cases, animal-based protein-rich foods may displace key nutrient-rich plant foods in dietary patterns where inadequate intake of fruits and vegetables has also been reported [11,13]. In particular, low intakes of dietary fiber characterize the diets of linemen [5,6,11,12], a group that may consume triple the amount of animal protein relative to plant protein [12]. Although NFL players typically experience decreased CVD risk and live longer than the general population [80], linemen experience increased CVD risk and mortality [81,82], making CVD the leading cause of death in the NFL [14].

It is also noteworthy that 33–67% of American football players reported daily use of protein supplements [11,12]. This contrasts with data from the AHS-2 cohort [33], which included limited capture of some protein supplements or meal-replacement shakes [59]. The top reported sources of protein consumption in participants following a vegan diet in this cohort included plant foods such as legumes and grains [59]. The modeled protein levels presented in this study are therefore attainable without protein supplementation and can be expected to meet the requirements of American football players. The effects of dietary protein on muscle protein synthesis have typically been studied through the use of supplemental, rather than whole food protein sources, and while these may differentially impact muscle protein remodeling, whole food sources of protein are recognized to effectively support muscular development and exercise recovery [83].

The results of the leucine modeling correlated with data from the Oxford arm of the European Prospective Investigation into Cancer and Nutrition (EPIC–Oxford), in which young men who followed completely plant-based diets reported consuming a similar proportion of calories from leucine [84]. Scaling to levels used in our study would result in leucine levels of 12.5–15.0 g/day, or 3.1–3.8 g/meal at each of four daily meals. Data have been published on the animal and plant protein contents of the diets of American football players [12], from which leucine levels can be projected using estimates that leucine comprises approximately 8.8% and 7.1% of these protein sources, respectively [52]. Reported intakes would yield 10.4–12.7 g/day of leucine, or 2.6–3.2 g/meal for each of four daily meals. These doses are sufficiently high such that additional leucine would be expected to produce no further gains in RET-induced muscle mass and strength [85,86]. Lysine and methionine levels were not modeled in this study. However, it has been proposed that consuming significant quantities of plant proteins, such as at the levels modeled in this study, is likely to overcome potential shortcomings that might otherwise limit the capacity of a plant-based diet to effectively support muscular development [87]. Levels of the remaining IAA were not calculated, because recommended intakes for maximal muscular development in response to RET have not been established. With regard to health, adherence to plant-based diets as consumed in developed countries has been argued to satisfy all amino acid requirements in the general population [88]. Scaling calorie and protein intakes to levels modeled in this study would further exceed these requirements.

Modeling of micronutrient adequacy indicated that recommendations for all vitamins and minerals were met with completely plant-based diets scaled to meet the energy needs of NFL players. Fiber, total omega-3 fatty acids, and linoleic acid values also met recommendations. Vitamin B12 has been identified as a nutrient of concern for populations following plant-based diets [89], and this study projected levels that surpassed recommendations (69.8 vs. 2.4 mcg/day) [53]. Vitamin D is another nutrient of concern in individuals following plant-based diets [60], although modeled values (average 755 IU/day) surpassed mean values reported in American football players, which were 152 and 184 IU/day for offensive and defensive players, respectively [12], and met recommendations of 600 IU/day [53]. Reliable sources of vitamins B12 and D include fortified foods and supplements, and are recommended to meet requirements [60]. There were seven micronutrients that exceeded the UL: folate, calcium, iron, magnesium, phosphorus, zinc, and sodium [54,55,56,57]. These values are unlikely to be problematic, due to factors such as the frequent establishment of UL based on supplemental versus dietary provisions and differences in bioavailability between animal- and plant-sourced micronutrients. Folate levels exceeded the UL (2353–2819 vs. 1000 mcg/day). However, this UL applies only to synthetic forms of folate obtained from supplements and fortified foods, whereas high intakes of folate from foods are not known to cause adverse effects [54]. Calcium levels surpassed the UL (3062–3670 vs. 2500 mg/day). However, the bioavailability of most plant-based sources of calcium is diminished due to the presences of oxalates and phytates found in these foods [90], and the majority of the adverse effects of hypercalcemia are related to supplementation [56]. Athletes also lose calcium through perspiration [91], and additional dietary calcium may offset these losses. Iron levels exceeded the UL (84–100 vs. 45 mg/day); the DRI for individuals adhering to vegetarian diets is 80% greater than the level set for individuals following omnivorous diets due to significant differences in the bioavailability of heme and non-heme iron [55], and it has been proposed that iron absorption fluctuates depending on the body’s iron stores and physiological needs [60]. Magnesium levels exceeded the UL (1728–2070 vs. 350 mg/day), but this UL applies only to pharmaceutical rather than food sources [57]. Phosphorus intake marginally surpassed the UL for every position except quarterback (4010–4353 vs. 4000 mg/day). However, phosphate concentrations are tightly regulated by the renal system [92] and it has been suggested that slight dietary excess is unlikely to cause harm to healthy individuals [93]. Zinc levels slightly exceeded the UL (43–52 vs. 40 mg/day), although it has been proposed that homeostatic mechanisms may alter zinc absorption and status in individuals adhering to vegetarian diets [60]. Sodium levels modeled in our study surpassed the recommended UL (10,578 versus 2300 mg/day).

Similar observations were made from survey data in American football players following omnivorous diets, whose mean sodium intakes were 4846–9404 mg/day [5,11,12]. Although these values significantly exceed guidelines, it is noteworthy that AHS-2 assessed sodium intake with an FFQ [33], a method which has demonstrated low reliability for estimating dietary sodium [94]. Athletes, especially those with higher sweat rates, also excrete more sodium and must therefore replace these losses through their diet [95]. Sodium losses in the sweat of NFL athletes range from 642–6700 mg/hour and, on days when players practiced for 4.5 h, calculated sodium losses were between 2.3 and 30 g/day [96]. A large body of evidence indicates that diets high in sodium significantly increase CVD risk [97], but the degree to which high-sodium diets affect the cardiovascular health of athletes with high sweat rates who engage in daily vigorous physical activity and excrete large quantities of sodium is unknown. Sodium intake guidelines for these populations must therefore consider individual differences in physiology and needs [11]. Finally, saturated fat values in this study represented approximately 5% of total calories, meeting the recommended upper limit of 10% [53]. In contrast, survey data from research into the diets of American football players have indicated mean saturated fat intakes between 10–13% [5,6,11,12]. These differences are significant due to the established association between saturated fat intake and CVD incidence [98] and mortality [99].

Our study has several strengths. First, in accordance with our prior modeling research in competitive male bodybuilders [23], dietary values were calculated using data from a large sample of individuals following plant-based diets [33]. Second, this cohort lived in the United States, the nation in which the NFL resides, increasing the potential for cultural compatibility of these athletes’ diets. Third, calorie requirements differ for athletes playing different positions [10]. Accordingly, protein values were modeled separately for these athletes to provide more granular data for each player position.

Our study also has limitations. One limitation inherent in our methodology is that we employed computational modeling to quantify dietary consumption instead of relying on direct laboratory methods, potentially introducing variations between anticipated and observed levels. For instance, dietary fiber levels of 140 g/day were calculated, yet fiber has been shown to increase satiety and large intakes may pose challenges for athletes with extreme energy expenditures striving to meet calorie needs [100,101,102]. In practice, this could elicit gastrointestinal disturbances and affect the composition and volume of meals selected and consumed by athletes. However, the digestive systems of athletes are adaptable and can facilitate greater energy intakes through expedited gastric emptying, reduced satiety, diminished bloating, greater tolerance to high volumes of food, and accelerated absorption rates [103]. Some of these adaptations are nutrient-specific. For example, high-carbohydrate diets have been proposed to increase the density and activity of sodium-dependent glucose-1 (SGLT1) transporters, which limit glucose transport, thereby facilitating additional absorption and oxidation of carbohydrates during exercise [103]. The completely plant-based diets consumed by the AHS-2 cohort were high in carbohydrates, with mean intakes comprising 62% of calories [33], indicating that this adaptation may be particularly relevant to the modeled diet. Nonetheless, recommendations have been made that athletes at risk of gastrointestinal distress consume low-fat, high-carbohydrate solid meals containing limited dietary fiber (e.g., oatmeal with fruit) hours before competition or drink liquid meals (e.g., fruit smoothie with soy milk) to meet energy needs without promoting digestive discomfort or excessive satiety [104]. Recommendations have also been made that athletes who adhere to plant-based diets and find it challenging to consume sufficient calories should increase their meal frequency and preferentially select energy-dense foods such as nuts and seeds, nut and seed butters, dried fruit, and hummus [72]. Similarly, the energy requirement calculations relied on published anthropometric measurements in American football players. These modeled results may differ from individual requirements, based on individual variations in body composition and activity levels. A second limitation is that this study did not directly address protein digestibility or amino acid availability, which are commonly considered poor in plant proteins. Interventional trials have aimed to overcome these challenges via supplying subjects with supplemental plant or animal protein concentrates and isolates that minimize anti-nutrient content and enhance bioavailability and have found no significant differences in their effects on absolute lean mass or muscle strength [29,105,106,107]. Protein supplements could result in significantly different MPS responses compared with protein from intact food sources [108]. However, the clinical relevance of this issue remains controversial because the most precise human data that assess real oro–ileal nitrogen digestibility indicate differences in the digestibility of animal and plant protein of just a few percent, despite marked differences in their content of fiber and other dietary constituents that can influence the digestion of proteins [88]. Furthermore, an increasing body of evidence suggests that protein consumption from whole food sources is effective in supporting gains in muscle mass, strength, and recovery from exercise, although more research is required to elucidate the comparative effects of whole food and supplemental protein sources [109]. Also, this study did not address protein quality, which is more influential for muscular development at lower protein levels than those modeled in this study [61]. Common methods for assessing protein quality such as the digestible indispensable amino acid score (DIAAS) also have notable limitations, especially in relation to plant-based diets [110], which diminish their application in this context. Instead, dietary leucine was modeled because a large body of evidence substantiates the ability of the leucine trigger hypothesis to explain a significant portion of the discrepant MPS rates following the consumption of different protein sources [111]. A third limitation is that our study did not model the ingestion of performance-enhancing compounds, such as creatine, that may differ between dietary patterns. A lack of dietary creatine has been proposed to limit the anabolic potential of plant-based diets [101,112,113,114,115,116], and we were unable to model these levels because creatine values have not been reported in large cohort studies of populations following plant-based diets [117]. However, while evidence suggests that supplemental creatine can augment RET-induced increases in muscular hypertrophy [118] and strength [119,120], it is unclear whether supplemental creatine improves exercise performance in athletes who avoid meat to a greater extent than in athletes consuming an omnivorous diet [121]. There is also a lack of evidence indicating that dietary creatine is capable of exerting these effects, potentially limiting its relevance in this modeling study [122]. Fourth, although our model included the majority of essential nutrients for which deficiencies are most prevalent [117], were unable to model levels of thiamin, riboflavin, niacin, pantothenic acid, biotin, chloride, iodine, sulfur, cobalt, copper, manganese, or selenium. Fifth, the degree to which dietary supplements contributed to nutrient adequacy is unknown, because nutrients from foods, beverages, and supplements were summed and only totals were presented. This study modeled dietary intakes in American football players following completely plant-based diets. Future research should model plant-based dietary habits among other athletic demographic groups such as women, as well as athletes participating in other sports, in whom levels of LBM have been shown to predict physical performance [123,124]. Prospective research comparing performance and changes in muscle mass among athletes eating plant-based versus omnivorous diets is also warranted.

## 5. Conclusions

This modeling study determined that completely plant-based diets scaled to meet the energy needs of American football players fulfilled protein, leucine, and essential micronutrient requirements. Our findings indicate that these diets satisfied dietary protein recommendations for optimal muscular development and athletic performance. Levels of leucine, a critical amino acid in the stimulation of MPS, also surpassed the threshold needed to maximize RET-induced anabolism. Moreover, this study underscores the capacity of scaled plant-based diets to satisfy micronutrient requirements to support health. Our results have considerable implications for public health and athletic performance, offering preliminary evidence that American football players who follow completely plant-based diets can meet their athletic objectives while aligning with broader health recommendations.

## Figures and Tables

**Table 1:** Energy requirements and modeled protein and leucine levels in American football players following completely plant-based diets.

Position	Body Mass (kg) [8,9]	Energy Requirements (kcal/Day) [10]	Absolute Protein Requirements (g/Day) [38]	Absolute Protein Levels (g/Day)	Relative Protein Levels (g/kg/Day)	Absolute Leucine Levels (g/Day) [52]	Leucine Levels per Meal (g)
Defensive Lineman	134.7	6250	216	227	1.7	16.1	4.0
Offensive Lineman	144.0	6350	230	230	1.6	16.3	4.1
Running Back	110.5	5850	177	212	1.9	15.1	3.8
Tight End	123.3	6150	197	223	1.8	15.8	4.0
Linebacker	108.1	6050	173	223	2.1	15.8	4.0
Quarterback	100.9	5300	161	219	2.2	15.5	3.9

g: grams; kg: kilograms.

**Table 2:** Modeled micronutrient and other key nutrient levels and requirements for American football players following completely plant-based diets, based on player position.

	AHS-2 Strict Vegetarian ‡	Defensive Lineman	Offensive Lineman	Running Back	Tight End	Linebacker	Quarterback	Nutrient UL	Nutrient Target	Recommendation	Target Met?
Calorie intake (kcal)	2000	6250	6350	5850	6150	6050	5300	-	-	-	N/A
Saturated fat (% kcal)	5.2	5.2	5.2	5.2	5.2	5.2	5.2	10	<10	DGA	✓
Total omega-3 (g)	2	6.3	6.4	5.9	6.2	6.1	5.3	ND	1.6	AI	✓
Linoleic acid (g)	20	61	62	57	60	59	52	ND	17	AI	✓
Fiber (g)	47	146	148	137	144	141	124	ND	74–89 †	DGA	✓
Vitamin A (RAE *)	1108	3463	3518	3241	3407	3352	2936	ND **	900	RDA	✓
Vitamin B6 (mg)	14.4	45	45.7	42.1	44.3	43.6	38.2	100	1.3	RDA	✓
Folate (mcg)	888	2775	2819	2597	2731	2686	2353	1000	400	RDA	✓
Vitamin B12 (mcg)	23.3	72.8	74	68.2	71.6	70.5	61.7	ND	2.4	RDA	✓
Vitamin C (mg)	531	1659	1686	1553	1633	1606	1407	2000	90	RDA	✓
Vitamin D (IU)	252	788	800	737	775	762	668	4000	600 IU	RDA	✓
Vitamin E (mg)	101	316	321	295	311	306	268	1000	15	RDA	✓
Calcium (mg)	1156	3613	3670	3381	3555	3497	3063	2500	1000	RDA	✓
Iron (mg)	32	99	100	92	97	96	84	45	8	RDA	✓
Magnesium (mg)	652	2038	2070	1907	2005	1972	1728	350	400	RDA	✓
Phosphorus (mg)	1371	4284	4353	4010	4216	4147	3633	4000	700	RDA	✓
Potassium (mg)	4234	13,231	13,443	12,384	13,020	12,808	11,220	ND	3400	AI	✓
Sodium (mg)	3531	11,034	11,211	10,328	10,858	10,681	9357	ND	2300	CDRR	✗
Zinc (mg)	16	51	52	48	50	49	43	40	11	RDA	✓

AI: adequate intake; AHS-2: Adventist Health Study 2; CDRR: chronic disease risk reduction level; DGA: ND: not determinable; RDA: recommended dietary allowance; RAE: retinol activity equivalent; UL: tolerable upper limit. Nutrient targets and recommendations sourced from the United States Department of Agriculture Dietary Guidelines for Americans (USDA DGA) 2020–2025 [53]. Nutrient UL derived from the National Academy of Medicine, formerly the Institute of Medicine [54,55,56,57]. * RDA for Vitamin A provided in RAE to account for different bioactivities of provitamin A carotenoids [54]. ** The UL for vitamin A is established only for preformed vitamin A (i.e., retinol, retinyl esters), which derive from animal-based foods, rather than carotenoids, which derive from plant-based foods [58]. The UL was therefore set as ND to reflect the dietary sources of the modeled cohort. † Based on recommendations to consume 14 g/1000 kcal. ‡ Calorie intake represents the estimated energy requirements needed to maximize muscle strength for athletic performance in American football players. Average micronutrient intakes per 2000 kcal/day reported in the AHS-2 dataset [33] were then scaled proportionally for each football player position.

## Data Availability

The original contributions presented in the study are included in the article.
